# Guidelines on analgosedation, monitoring, and recovery time for flexible bronchoscopy: a systematic review

**DOI:** 10.1186/s12890-021-01532-4

**Published:** 2021-06-10

**Authors:** Daniel Strohleit, Thomas Galetin, Nils Kosse, Alberto Lopez-Pastorini, Erich Stoelben

**Affiliations:** Lung Clinic Cologne-Merheim, Thoracic Surgery, Hospital of Cologne, University of Witten/Herdecke, Ostmerheimer Str. 200, 51109 Cologne, Germany

**Keywords:** Bronchoscopy, Guideline, Recovery time, Monitoring, Patient safety, Hypercapnia, Capnometry

## Abstract

**Background:**

Patients undergoing bronchoscopy in spontaneous breathing are prone to hypoxaemia and hypercapnia. Sedation, airway obstruction, and lung diseases impair respiration and gas exchange. The restitution of normal respiration takes place in the recovery room. Nonetheless, there is no evidence on the necessary observation time. We systematically reviewed current guidelines on bronchoscopy regarding sedation, monitoring and recovery.

**Methods:**

This review was registered at the PROSPERO database (CRD42020197476). MEDLINE and awmf.org were double-searched for official guidelines, recommendation or consensus statements on bronchoscopy from 2010 to 2020. The PICO-process focussed on adults (Patients), bronchoscopy with maintained spontaneous breathing (Interventions), and recommendations regarding the intra- and postprocedural monitoring and sedation (O). The guideline quality was graded. A catalogue of 54 questions was answered. Strength of recommendation and evidence levels were recorded for each recommendation.

**Results:**

Six guidelines on general bronchoscopy and three expert statements on special bronchoscopic procedures were identified. Four guidelines were evidence-based. Most guidelines recommend sedation to improve the patient’s tolerance. Midazolam combined with an opioid is preferred. The standard monitoring consists of non-invasive blood pressure, and pulse oximetry, furthermore electrocardiogram in cardiac patients. Only one guideline discusses hypercapnia and capnometry, but without consensus. Two guidelines discuss a recovery time of two hours, but a recommendation was not given because of lack of evidence.

**Conclusion:**

Evidence for most issues is low to moderate. Lung-diseased patients are not represented by current guidelines. Capnometry and recovery time lack evidence. More primary research in these fields is needed so that future guidelines may address these issues, too.

**Supplementary Information:**

The online version contains supplementary material available at 10.1186/s12890-021-01532-4.

## Background

Flexible bronchoscopy (FB) can be performed with various regimes of monitoring and sedation [1–3]. Non-invasive blood pressure, peripheral pulse oximetry and electrocardiogram are common practice for monitoring during bronchoscopy. Capnometry is not commonly performed. However, pulse oximetry is not adequate to identify apnoea phases during bronchoscopic procedures properly; particularly when patients receive supplemental oxygen during the procedure, the saturation may appear good and mask apnoea and hypoventilation [4].

Apnoea phases and resulting hypercapnia can therefore not adequately be detected. One third of the hypercapnic episodes appear delayed up to one hour after the end of FB [5]. Patients with pre-existing lung diseases (e.g. COPD) are even more at risk to develop substantial hypercapnia during and after FB [5, 6]. Thus, the patients need to be monitored after FB, too, to determine the safe time of discharge in ambulant patients or of transfer to the ward in hospitalised patients.

This adequate time also depends on the type and depth of sedation. Sedative regimes range from no sedation to general anaesthesia, although there is evidence that the patient’s comfort is higher if FB is performed in analgosedation [2]. While rigid bronchoscopy is mostly performed under general anaesthesia to facilitate complex procedures (for example, airway stent placement or foreign body extraction), FB is often performed under sedation with preserved spontaneous breathing [7]. Most commonly benzodiazepines (midazolam), propofol and opioids (alfentanil, remifentanil and fentanyl) are used for sedation in FB. There is no standard practice of sedation for FB, as almost every combination of sedative drugs is acceptable; mostly the type of sedation depends on the discretion of the examiner [3].

Although the depth of sedation impacts oxygen saturation, carbon dioxide tension, and recovery time, we hardly found any evidence or recommendations on the issues of monitoring, capnometry and observation time in a preliminary research. Thus, there is a need for elucidating the current evidence, so we conducted a systematic review of the current bronchoscopy guidelines on flexible bronchoscopy with focus on these topics.

## Methods

### Study design

This systematic review is registered on the PROSPERO database (www.crd.york.ac.uk/prospero/CRD42020197476) and is performed following the PRISMA-P reporting guidelines (Preferred Reporting Items for Systematic Reviews and Meta-Analyses Protocols [8]). The completed PRISMA-P checklist is provided in Additional file 1: Table S1.

Clinical questions were gathered in the PICO (Patient, Intervention, Control, Outcome) format to define the scope of the guideline and inform the literature search: The population under review compasses adult subjects, the intervention is any bronchoscopic procedures with maintained spontaneous breathing, the comparators are guidelines, recommendations, or consensus statements. The main outcomes are the recommendations regarding the intra- and postprocedural monitoring and sedation.

### Search strategy

The systematic search is performed in the electronic databases Medline (using Pubmed) and awmf.de on July 06 2020. The AWMF ("Arbeitsgemeinschaft der Wissenschaftlichen Medizinischen Fachgesellschaften e.V." or “Association of the Scientific Medical Societies in Germany”) publishes official German guidelines of 175 scientific member societies and 3 associated societies from all medical specialties. The details of the systematic search are provided in the supplementary material.

### Study selection

An article was considered eligible if it: (1) is an official guideline, recommendation or consensus statement of a national or international medical institution; (2) is evidence- or consensus-based; (3) recommends on the practice of bronchoscopy with maintained spontaneous breathing; (4) was published within the last ten years; (5) was presented in full-text form and in English or German language. Clinical trials, case series and reports, expert opinions, teaching literature, meta-analyses, and systematic reviews were excluded.

Records were managed by electronic citation managers to screen the results of the database research. The search results were screened for eligibility based on title and abstract by two reviewers (DS and TG). Subsequently, full-text articles were evaluated on relevance by the same reviewers. Studies, which do not give practice recommendations on how sedation, monitoring, and observation should be performed, were excluded. The reasons for the exclusion of an article were documented. Disagreements were resolved through discussion and consensus; if an agreement could not be achieved, the decision was made by the senior researcher (ES).

### Data extraction

The following data were extracted from all included studies: main author’s last name; year of publication; publishing institution (stakeholder); type of guideline (consensus or evidence-based); patient characteristics; bronchoscopic intervention; practice recommendations on monitoring and sedation, the strength of recommendation; the level of evidence. Data are entered into a catalogue of 54 items (Table 1), which are organised in eight major topics: the monitoring during FB, sedation and local anaesthesia, sedative drugs, analgesic drugs, termination of examination, patients with pre-existing lung-diseases, the management of hypoxemia, the monitoring after FB and the recovery time after FB.

**Table 1:**
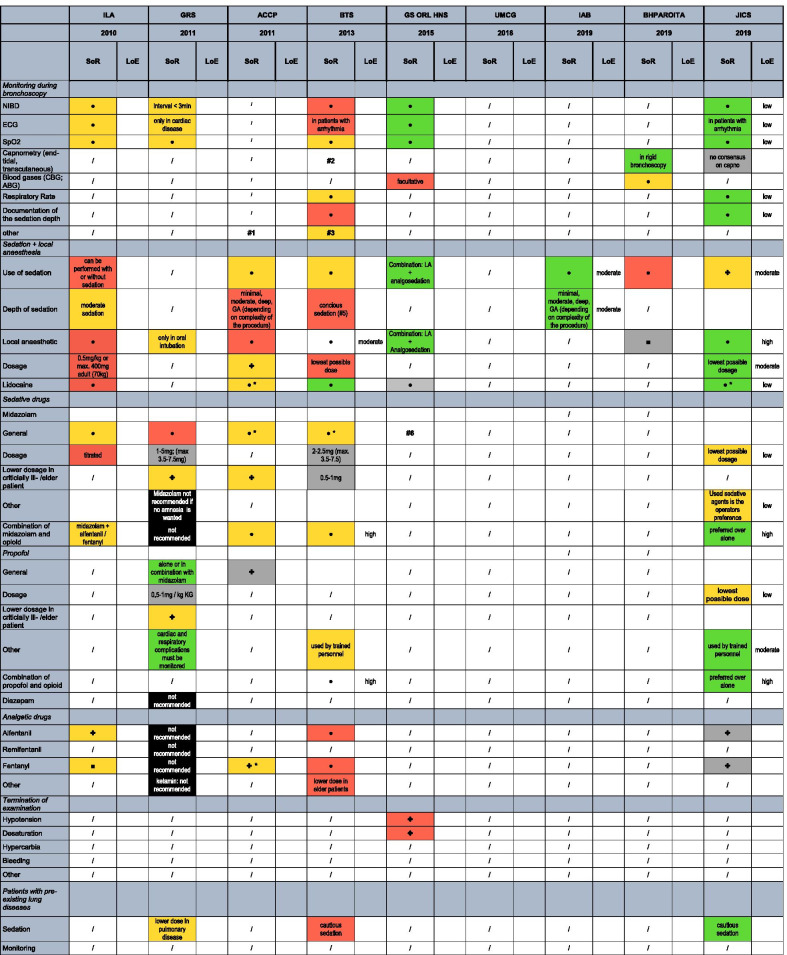

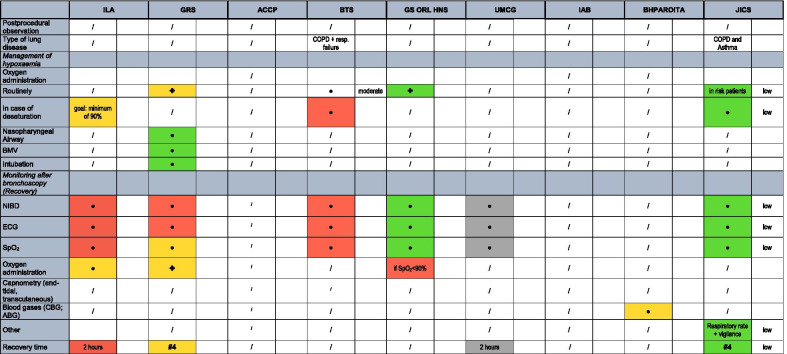
Overview of recommendations, strength of recommendation and levels of evidence of current guidelines on bronchoscopy

SoR, strength of recommendation; LoE, level of Evidence. Legend: ● yes; ✤ yes, but not specified; * preferred substance; red: weak, yellow: moderate, green: strong recommendation; black: recommendation against item; grey: item discussed, but no recommendation due to lack of evidence. Footnotes: #1: citation from guideline “For the purpose of this document, adequate monitoring of the level of consciousness and physiologic variables (including BP, respiratory rate, oxygen saturation by pulse oximetry, and ECG monitoring) is assumed and will not be further discussed.” #2: citation from guideline: "Continuous multimodal physiological monitoring should be undertaken during and after bronchoscopy in the ICU setting." #3: citation from guideline: "Patients who are more deeply sedated should have the same level of care monitoring as in general anesthesia." #4: Patients remain in the recovery room until preprocedure level of consciousness and acceptable vital parameters are reached. #5: Recommendations for sedation are based on the „S3-guideline Sedation in gastroenterologic endoscopy “ (1)

The systems to rate the level of evidence and the strength of recommendation were gathered and re-assigned to three levels as shown in Tables 2 and 3.

**Table 2:**
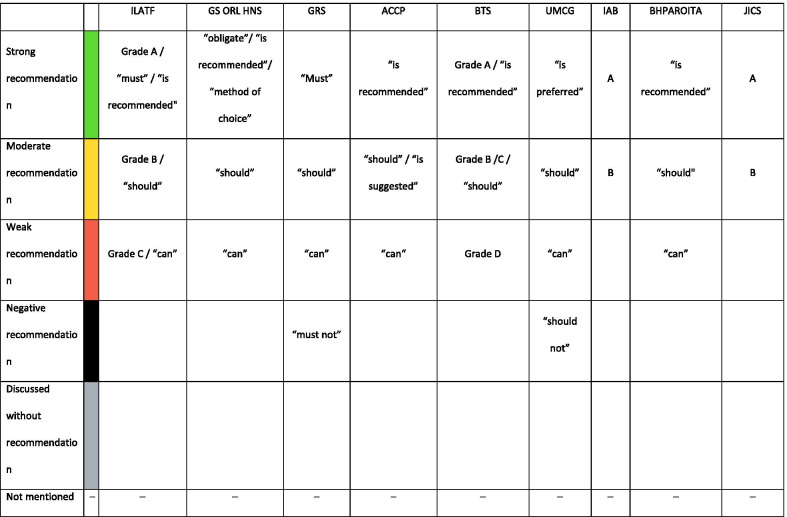
Strength of recommendation

UPP, usual practice point (GRADE System)

**Table 3:** Levels of evidence

Level of evidence	ILATF	BTS	IAB	JICS
High Evidence	**GRADE A:** requires at least one randomized control trial as part of a body of literature of overall good quality and consistency addressing the specific recommendation	**GRADE A/B** | 1 + + and 1 +	“**Level 1:** High‐quality evidence supported by findings from well‐executed randomized controlled trials or unequivocal evidence from well‐conducted observational studies with strong effects”	"**Level 1:** High‐quality evidence supported by findings from well‐executed randomized controlled trials or unequivocal evidence from well‐conducted observational studies with strong effects "
Moderate Evidence	**Grade B:** requires availability of well-conducted clinical studies but no randomized clinical trials on the topic of the recommendation	**GRADE C/D** | 1-/2 + /2 + +	**Level 2**: Moderate‐quality evidence from randomized trials or from several observational studies with some limitations (inconsistency, indirectness, flaws in conduct, reporting bias, imprecise estimates, small sample size, or others)	**Level 2:** Moderate‐quality evidence from randomized trials or from several observational studies with some limitations (inconsistency, indirectness, flaws in conduct, reporting bias, imprecise estimates, small sample size, or others)
Low Evidence	**"Grade C**: requires evidence from expert committee reports or opinions and clinical experience of respected authorities."	**Important practice point** | 3 and 4	**"Level 3:** Low‐quality evidence from observational studies or from controlled trials with serious limitations UPP: Not supported by sufficient evidence; however, a consensus reached by the working group, based on clinical experience and expertise "	"**Level 3:** Low‐quality evidence from observational studies or from controlled trials with serious limitations UPP: Not supported by sufficient evidence; however, a consensus reached by the working group, based on clinical experience and expertise "

### Risk of bias

To assess the risk of bias, the AGREE II tool was used [9]. The AGREE II tool assesses the quality of a guideline by checking 23 items organized within six domains, with each domain capturing a specific aspect of guideline quality:

1. scope and purpose (three items)
2. stakeholder involvement (three items)
3. rigor of development (eight items)
4. clarity of presentation (four items)
5. applicability (three items)
6. editorial independence (two items)

Items are rated on a seven-point scale from 1 (strongly disagree) to 7 (strongly agree). A quality score is calculated for each of the six domains, presented as the percentage of the maximum possible score for each specific domain.

We used a staged scoring process to assess the quality of the included guidelines as proposed by the 2017 AGREE-II-manual [10]: First, we chose the guidelines which cover general bronchoscopic procedures according to our PICO. Expert panels on a narrow subtopic (for example, only cryobiopsies) were not included because of their limited contribution to the main question of this review. Second, one reviewer (DS) assessed the third domain subscale (rigor of the development) of all guidelines. Third, the guidelines with high scores on this domain (e.g. ≥ 70%) were evaluated by four reviewers (DS, TG, NK, AL) on all domains.

## Results

### Included guidelines

**Fig. 1:**
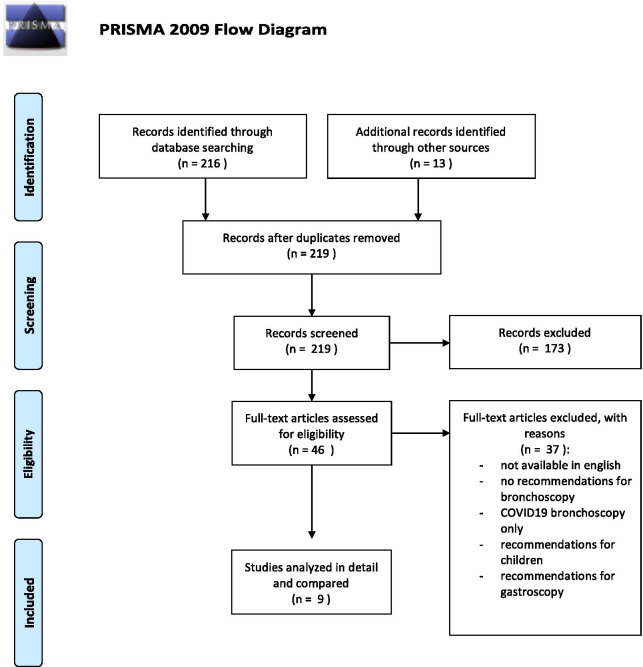
PRISMA flow chart of the process of identifying eligible guidelines

Forty-four guidelines, recommendations and consensus paper were identified, of which 37 had to be excluded (Fig. 1): Twenty-five papers did not give recommendations specific for bronchoscopy, two focussed on gastroscopy [11, 12], two referred to bronchoscopy in children [13, 14], two papers only dealt with the technical aspect of EBUS [15, 16] and two papers dealt with precautions in bronchoscopy of COVID19-patients [17, 18]. Four papers were excluded because they were only available in Chinese [19–22]. All other guidelines were available in an English version.

Nine guidelines, expert recommendations and consensus statements met the eligibility criteria and were included. They are listed in Table 4 and can roughly be sorted by their scope: four guidelines and two consensus papers cover recommendations for sedation, monitoring and analgesia of general bronchoscopy (in chronologic order: ITALF, GRS, ACCP, BTS, GS ORL HNS, JICS). Three expert panels focus on special bronchoscopic procedures (UMCG: coils for endoscopic lung volume reduction; IAB: lung cryobiopsies; China: the treatment of central airway stenosis) and add limited information relevant to this review.

**Table 4:** Included guidelines, recommendations and consensus paper sorted by publication date

Region	Year	Society/Institution	Acronym	First author	Type of statement	Scope
Israel	2010	Israel Lung Association Task Force	ILATF	Shulimzon [23]	Guideline	General bronchoscopy
Germany	2010	German Society of Oto-Rhino-Laryngology and Head-and-Neck-Surgery	GS ORL HNS	Schmidt [24]	Guideline	General bronchoscopy
Germany	2011	German Respiratory Society	GRS	Hautmann [25]	Consensus paper	General bronchoscopy
USA	2011	American College of Chest Physicians	ACCP	Wahidi [26]	Consensus paper	General bronchoscopy
UK	2013	British Thoracic Society	BTS	Du Rand [27]	Guideline	General bronchoscopy
Netherlands	2018	Department of Pulmonary Diseases, University of Groningen, University Medical Center Groningen, Groningen, The Netherlands	UMCG	Slebos [28]	Expert recommendation	Endoscopic lung volume reduction
India	2019	Indian Association for Bronchology	IAB	Dhooria [29]	Expert recommendation	Cryobiopsy
China	2019	Beijing Health Promotion Association Respiratory and Oncology Intervention and Treatment Alliance	BHPAROITA	Jin [30]	Expert recommendation	Malignant central airway stenosis
India	2019	Joint Indian Chest Society/National College of Chest Physicians (I)/Indian Association for Bronchology	JICS	Mohan [31]	Guideline	General bronchoscopy

**Fig. 2:**
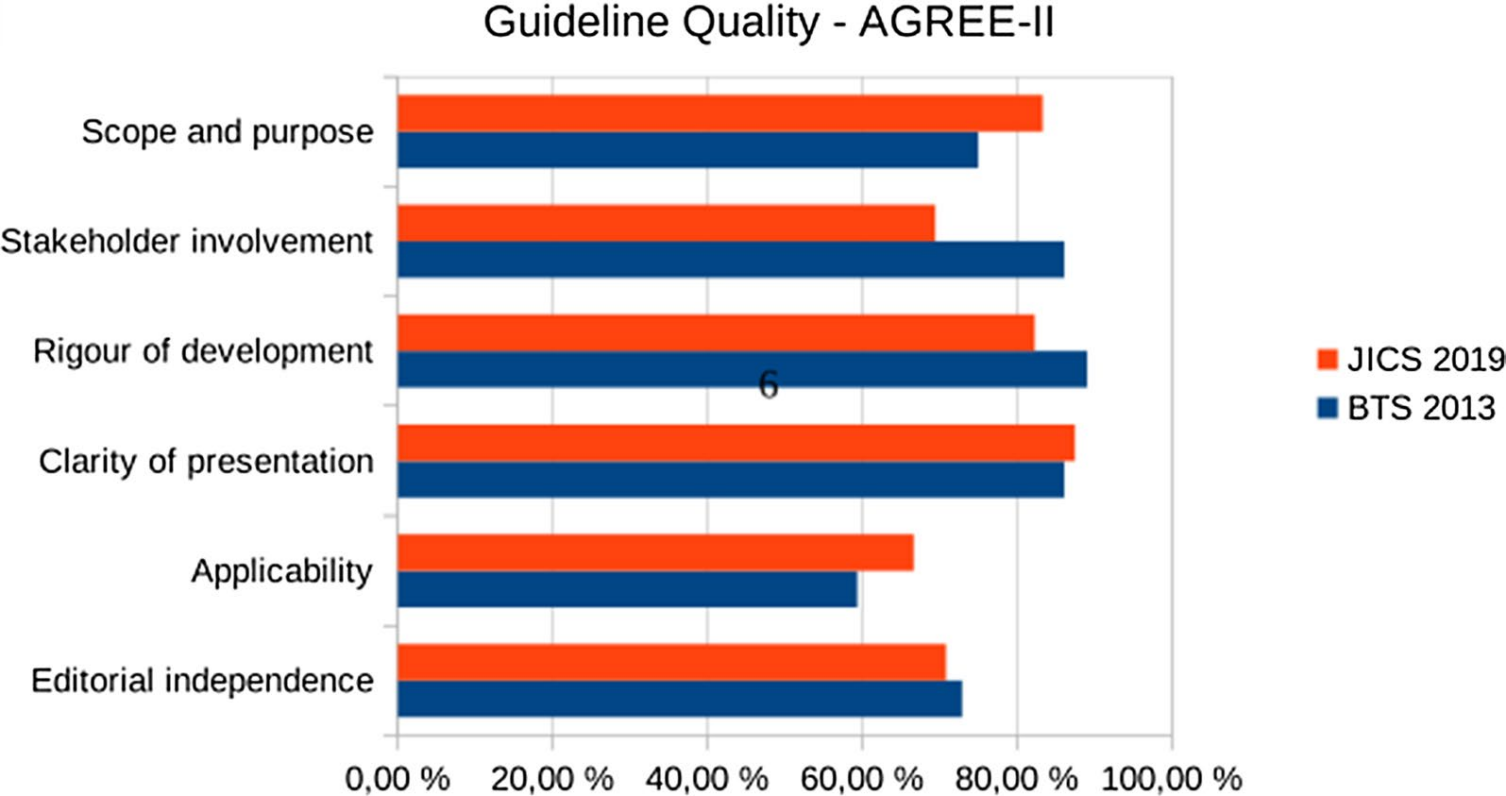
AGREE II assessment for high-quality guidelines on general bronchoscopy

The six general guidelines were rated for the rigour of development with the AGREE-II-tool as follows: ITALF 31%, GRS 21%, ACCP 40%, BTS 100%, GS ORL HNS 31%, JICS 85%. The detailed rating of the BTS and JICS guidelines, which reached > 70% in the domain “rigour of development”, is presented in Fig. 2.

### Evidence levels and strength of recommendation

Four guidelines are evidence-, five are consensus-based. Three different systems to rate the levels of evidence are used (Table 3). Most evidence levels are low or moderate, but over time some of them improve to high evidence levels (Table 1). Six papers are grading their strength of recommendation, of which four use a modified GRADE system and four use the phrasing, for example, “should”, “can” or “must” (Table 2).

### Monitoring during FB

Non-invasive blood-pressure (NIBP) measurement, peripheral pulse oximetry (SpO_2_) and electrocardiogram (ECG) were recommended by five studies, with a strength of recommendation ranging from weak to strong recommendations, with an overall low evidence level. The JICS guideline of 2019 strongly recommend NIBP and SpO_2_ with a low level of evidence. The use of an ECG during FB is only strongly recommended in patients with cardiac arrhythmias. Furthermore, the JICS endorse that the respiratory rate and the depth of sedation should be documented during FB. The ACCP assume adequate monitoring for FB and does not discuss it further.

### Capnometry

Capnometry, whether end-tidal, transcutaneous or a measurement of partial carbon-dioxide pressure in capillary blood gas analysis, is not covered by the guidelines. The BTS mentions “continuous multimodal physiological monitoring during and after FB in the ICU setting” and “patients who are more deeply sedated should have the same level of care monitoring as in general anaesthesia”, but does not explicitly name capnometry as part of the monitoring. The JICS did not find a consensus regarding the use of capnometry, although sedative agents may induce hypercarbia during sedation. The use of capnometry was not mentioned for the post-procedural observation time. The BHPAROITA recommends blood gas analysis during and after bronchoscopy in patients with malignant central airway stenosis.

### Local anaesthesia and sedation

A local anaesthesia (LA) is recommended by 4 studies, with one strong, one moderate and two weak recommendations. According to the BTS, LA reduces cough, provides a better patient tolerance and may reduce the required doses of sedative agents (moderate evidence level). The GRS gives a moderate recommendation for the usage of a LA if oral intubation is performed. More recent guidelines find more evidence for the use of LA and give stronger recommendations than the older ones. Four studies recommend lidocaine for LA, two of which as preferred agent. The strengths of recommendation for lidocaine increase from 2010 to 2019.

The use of sedation for FB is discussed by seven studies. Six studies recommend the use of sedation for FB with strength of recommendation ranging from weak to strong. The ILATF and BTS say that bronchoscopy without analgosedation can be performed as well, but patients’ preference should be sought. Evidence levels are low or moderate. Two guidelines recommend a moderate or conscious sedation, two propose to adapt the depth of sedation to the complexity of the procedure, ranging from minimal sedation to general anaesthesia.

### Sedative drugs

#### Midazolam

Four guidelines recommend Midazolam, two of which as the preferred sedative agent. Three guidelines recommend using the lowest possible dose. Lower dosage of midazolam in particular patient groups (elder patients/ critically-ill patients) were endorsed by two studies.

#### Propofol

The GRS 2011 recommends propofol either as mono-sedation or combined with midazolam. In contrast, the JICS and BTS endorse the combination of propofol with an opioid over propofol alone to improve the patient’s tolerance, supported by a high level of evidence. The lowest possible dose of propofol should be applied Propofol should only be given if the personnel are trained in the administration of propofol. According to the JICS, propofol must be cautiously administered in high-risk patient groups.

#### Diazepam

According to the GRS, diazepam should not be used because of its long half-life. The other guidelines do not mention diazepam.

### Analgesic drugs

The combination of midazolam and opioids is recommended by four guidelines, particularly to improve the patient’s tolerance during FB and to reduce the total dosage of sedative agents. Only the 2011 GRS guideline recommends against the use of opioids because of the risk of hypoventilation and they “have no advantages over the preferred substances [i.e. midazolam and propofol]”.

### Patients with pre-existing lung diseases

Recommendations for pre-existing lung diseases were only covered by three papers regarding sedation. The BTS weakly recommends a cautious sedation of patients with COPD or respiratory failure. The GRS gives a moderate recommendation that a lower dosage of sedative agents should be used in pulmonary disease. The JICS gives a strong recommendation of cautious sedation for patients with COPD and Asthma. An extended monitoring or post-procedural observation time for patients with pre-existing lung diseases is not discussed by the guidelines.

### Management of hypoxemia

Four guidelines endorse the routine administration of oxygen during FB (low to moderate evidence), three guidelines limit oxygen to certain cases (risk patients, desaturations below 90% or for more than one minute). An explicit statement for the stepwise escalating management for persistent hypoxemia was not given by the guidelines. Advanced airway equipment must be present according to the GRS.

### Monitoring after FB

Advice for post-procedural monitoring, particularly NIBP, SpO_2_ and ECG is given by five guidelines. The strengths of recommendation increase until 2019, but the level of evidence remains low. The GRS mentions SpO_2_ as the minimum monitoring. The UMCG mentions monitoring of NIBP, SpO_2_ and ECG, but gives no recommendation. The JICS endorses the monitoring of vigilance and respiratory rate after FB.

### Recovery

Regarding the time patients are monitored in the recovery room, only the ILATF gives a specific recommendation of 2 h. One guideline mentions the recovery time of 2 h, but without a recommendation. Two guidelines propose, without going into detail, that patients be observed until they reach a level of pre-procedure consciousness and acceptable vital parameters.

## Discussion

This systematic review is the first work to focus on the issues of monitoring and recovery in bronchoscopies.

Insufficient monitoring combined with too deep sedation and undetected respiratory depression can lead to substantial complications in bronchoscopic procedures [32]. Hence, it is important to define the adequate kind of monitoring. The use of NIBP, heart rate and pulse-oximetry is common practice in FB as stated by six guidelines with mostly moderate to strong recommendations. However, only low to moderate evidence exists regarding its use. An electrocardiogram is routinely advised only by two guidelines in 2010 and 2011; more recent guidelines (BTS, JICS) propose an ECG only in patients with known cardiac disease because the incidence of arrhythmia in FB is reported to be very low (0.02%, low level of evidence) [33, 34]. The practice guidelines for moderate sedation and analgesia by the American Association of anaesthesiologists align with the recommendation for the use of ECG in patients with cardiac risk history [35].

Beside circulation, respiration is the main system to take care for during bronchoscopy. For instance, pulse oximetry is an adequate method to monitor hypoxemia during bronchoscopy [33, 36]. Nonetheless, the detection of hypoxemic episodes and apnoea phases may be impaired or delayed in patients receiving supplemental oxygen during the procedure [4, 37], which is routinely advised for by some guidelines. The majority of current guidelines recommend the use sedative and analgesic substances for flexible bronchoscopy, accepting the drug-induced respiratory depression. The alveolar hypoventilation does not only result in hypoxemia, but also in hypercarbia. Consequently, capnometry during flexible bronchoscopy can lead to the earlier detection of apnoea phases, by approximately half a minute [38].

Hypercapnia is not only a predictor of apnoea, but leads to consciousness disorders and hypotension [39], particularly in patients with pre-existing lung diseases [6, 40]. They reach higher levels of carbon dioxide and need more time to recover to normocapnia [41]. Nonetheless, continuous measurement of carbon-dioxide tension during or after FB in sedation is not mentioned by one of the guidelines reviewed here. The JICS finds no consensus regarding the role of capnometry, although they recognise the problem of drug-induced hypercapnia. The JICS base their statement on three literature references [42–44], one of which strongly endorses capnography during procedural sedation and analgesia with a high level of evidence. The cited ASA reference of 2002 [43] has been replaced meanwhile in 2018, and now also recommends the use of end-tidal capnography during moderate sedation to reduce the number of hypoxemic events [35]. The Association of Anaesthetists of Great-Britain and Ireland recommends capnography monitoring for anaesthetised patients after anaesthesia until a full level of consciousness is reached if they were deeply sedated [44]. Furthermore, a meta-analysis of 2011 showed that respiratory depression has been detected more often with the use of capnometry, compared to standard monitoring [45].

However, the evidence which the bronchoscopy guidelines as well as the ASA and ESA (European Society of Anaesthesiology) guidelines on sedation rely on, mainly rises from studies on gastrointestinal endoscopies [46–48]. In bronchoscopy, the bronchoscope itself placed in the airways causes an obstruction, which makes hypercapnia and hypoxemia even more likely to occur than in gastrointestinal endoscopy [49]. Most of these studies use end-tidal capnometry to detect apnoea phases earlier during sedation, but end-tidal capnometry can be insufficient to measure carbon-dioxide tension properly in the case of a ventilation perfusion mismatch.

Particularly in patients with pre-existing lung diseases or obstructive sleep apnea, transcutaneous capnometry is more precise in detecting hypercapnia during and after procedures with maintained spontaneous breathing than end-tidal capnometry [50]. However, the values of transcutaneous capnometry appear delayed and do not provide a real-time assessment of the respiratory function [51, 52]. Transcutaneous capnometry is further impaired by a technical drift, hypoperfusion of the skin, improper calibration, and air bubbles under the sensor [52].

In all the investigated guidelines of this review, no recommendation was given on the management of hypercapnia or a tolerable threshold of partial pressures of carbon dioxide during FB.

Even though there is no evidence for tolerable thresholds of carbon dioxide during the procedure, after which the procedure should be immediately terminated, capnometry could help to identify patients at risk. Especially in long lasting procedures, such as EBUS or endoscopic lung volume reduction, capnometry can indicate a longer or more intensive postoperative observation [41, 53]. In patients with a high risk for alveolar hypoventilation, hypoxemia, and hypercapnia during FB, non-invasive ventilation (NIV) can improve ventilation. The BTS states that NIV may be considered in intensive care patients with preprocedural hypoxemia [27]. A systematic review has shown a non-significant trend that high-risk patients with FB under NIV are less likely to suffer from a “postprocedural delayed respiratory failure” [54].Thus, NIV is a feasible alternative to facilitate FB in these patients.

The type and depth of sedation influence the necessary recovery time, too [2, 42, 55]. The use of sedation has changed over time, as well as the sedative agents used in flexible bronchoscopy. While relinquishing sedation was recommended as a possible option for bronchoscopy by ILATF in 2010 [23], actual guidelines recommend the use of sedative agents to improve patients’ tolerance [31]. This review could not identify a uniform statement regarding the desired depth of sedation for FB. The recommendations range from minimal to moderate or conscious sedation as well as deep sedation or general anaesthesia. A more complex procedure implies a deeper sedation [27]. Nevertheless, guidelines suggesting a feasibility of FB without sedation did not take patient-relevant endpoints into account, such as procedure tolerance and therapy adherence [56]. Sedation during FB enhances the willingness of patients to repeat the procedure and their comfort during the procedure [3, 57–59]. Furthermore, it reduces the duration of the bronchoscopy [60].

A trend could be identified regarding the sedative agents: While midazolam alone was preferred in the earlier guidelines, the combination of sedative agents with opioids is favoured now. The combination reduces cough, pain (due to the insertion of the endoscope), improves the patients’ tolerance, and reduces the total dose of sedative agents (high level of evidence). Furthermore the use of topical anaesthesia reduces cough and is beneficial for the patients tolerance [61]. As explained above, the use of sedation and opioids may provoke hypoxaemia and hypercapnia. The reviewed guidelines did not deal with the question, when to prematurely terminate a bronchoscopy in the case of critical incidents. Although the incidence of complications during FB and resulting morbidity is low [34, 62], future guidelines should address thresholds or necessary interventions to manage complications of FB [63].

Four guidelines concern the issue of the appropriate recovery time, two of which recommend two hours of observation, and two recommend keeping the patient in the recovery room until the preprocedural level of consciousness and acceptable vital parameters are reached; these statements are not further specified. Neither the quantitative (“two hours”) nor the qualitative approach are supported by evidence. The ASA states that there is insufficient literature concerning the appropriate monitoring, as well as recovery time and discharge criteria during recovery care. A vague recommendation is given to monitor the patients’ oxygenation and circulation until a pre-procedure level of consciousness is re-attained, which aligns with the reviewed guidelines [35].

None of the guidelines try to relate their recommendations on recovery time to patient-related factors (comorbidities, obesitas), the type of bronchoscopy, or the type or depth of sedation. From our point of view, it is desirable to focus on these issues, because adequate recommendations could help to safely discharge of patients from the recovery unit and simultaneously limit the necessary resources to a sensible extent.

## Conclusion

The recommendations in the reviewed guidelines resemble in content but differ in the strength of recommendations. For most issues, the underlying evidence is low or moderate. The topics capnometry and recovery time are not sufficiently covered. There are only few recommendations adapted to patients with chronic lung diseases.

Future guidelines for flexible bronchoscopy should include these issues in their scope and literature search. More primary studies on these topics are necessary.

## Supplementary Information

**Additional file 1**. PRISMA-P checklist.

## Data Availability

The resources to reproduce our results are all given in the reference list.

## Abbreviations

ACCP: American College of Chest Physicians
ASA: American Society of Anesthesiologists Physical Status Classification System
AWMF: Association of the Scientific Medical Societies in Germany
BHPAROITA: Beijing Health Promotion Association Respiratory and Oncology Intervention and Treatment Alliance
BTS: British Thoracic Society
COPD: Chronic obstructive pulmonary disease
EBUS: Endobronchial ultrasound
ECG: Electrocardiogram
ESA: European Society of Anaesthesiology
FB: Flexible bronchoscopy
GRS: German Respiratory Society
GS ORL HNS: German Society of Oto-Rhino-Laryngology and Head-and-Neck-Surgery
IAB: Indian Association for Bronchology
ICU: Intensive care unit
ILATF: Israel Lung Association Task Force
JICS: Joint Indian Chest Society
LA: Local anaesthesia
NIBP: Non-invasive blood pressure
PICO: Patient Intervention Control Outcome
PRISMA-P: Preferred Reporting Items for Systematic Reviews and Meta-Analyses Protocols
SpO_2_: Peripheral pulse oximetry
UMCG: University Medical Center Groningen
